# Ten simple rules for teaching practical AI to health data teams

**DOI:** 10.1371/journal.pcbi.1014915

**Published:** 2026-10-30

**Authors:** Eric Just, Aik Choon Tan

**Affiliations:** 1 Just Health Technologies, LLC, Salt Lake City, Utah, United States of America; 2 Department of Oncological Sciences, School of Medicine, University of Utah, Salt Lake City, Utah, United States of America; 3 Office of Data Science, Huntsman Cancer Institute, University of Utah, Salt Lake City, Utah, United States of America; Dassault Systemes BIOVIA Ltd, UNITED STATES OF AMERICA

## Abstract

Artificial intelligence (AI) has rapidly entered the healthcare discourse, often framed through the lens of frontier models, generative systems, and transformative potential. Yet for many healthcare data teams, the practical path from curiosity to capability remains unclear. Concerns about data privacy, regulatory compliance, brittle systems, and misaligned expectations frequently stall progress before meaningful experimentation can begin. This paper presents ten simple rules for teaching practical AI to healthcare data teams, grounded in real-world data, privacy-first experimentation, and system-level thinking. Drawing on hands-on training experiences and existing educational and methodological literature, we argue that AI education in healthcare must prioritize workflows over models, systems over components, and institutional capability over isolated technical success. These rules are intended to help educators and organizational leaders move teams beyond the AI hype cycle toward building trustworthy, sustainable, and context-aware AI systems that matter.

## Introduction

In recent years, headlines and boardroom conversations alike have been dominated by the rapid advance of artificial intelligence (AI) [1]. Breakthroughs in large language models (LLMs) and generative AI systems [2] have created a sense of urgency across industries, including healthcare, to “do AI.” Yet beneath this enthusiasm lies a persistent gap between aspiration and execution [3]. Healthcare data teams are often asked to adopt AI in environments characterized by fragmented data, strict privacy requirements, and high clinical stakes. In many organizations, this fragmentation is exacerbated by routing AI initiatives through small, elite teams of data scientists, without meaningfully engaging the broader teams responsible for developing the data infrastructure and analytic context required for scalable AI systems.

This tension is particularly acute in healthcare. Data are heterogeneous, frequently unstructured, and deeply contextual. Pathology reports, clinical notes, imaging metadata, and registry extracts rarely resemble the clean datasets used in tutorials or competitions. At the same time, regulatory and ethical constraints make indiscriminate use of cloud-based AI services infeasible for early-stage learning. As a result, many teams oscillate between overconfidence driven by demonstrations and paralysis driven by perceived risk.

This paper is about bridging that gap. Rather than focusing on specific models or rapidly evolving tools, we describe an approach to AI education that emphasizes foundational principles: how AI systems ingest, transform, retrieve, rank, and synthesize information; how different model architectures serve distinct roles within those systems; and how teams can experiment safely and realistically within their own institutional constraints. By grounding instruction in real healthcare workflows, privacy-first experimentation, and structured opportunities for applied practice, we aim to equip healthcare data teams to move beyond the hype cycle and become confident, independent builders and evaluators of trustworthy AI systems [3].

The ten rules presented here are ordered deliberately, progressing from foundational concepts to system design, governance, institutionalization, and feedback to improve teaching content for new batch of trainees. Together, they offer a practical canon for educators and leaders seeking to develop durable AI capability in healthcare settings (Fig 1).

**Fig 1:**
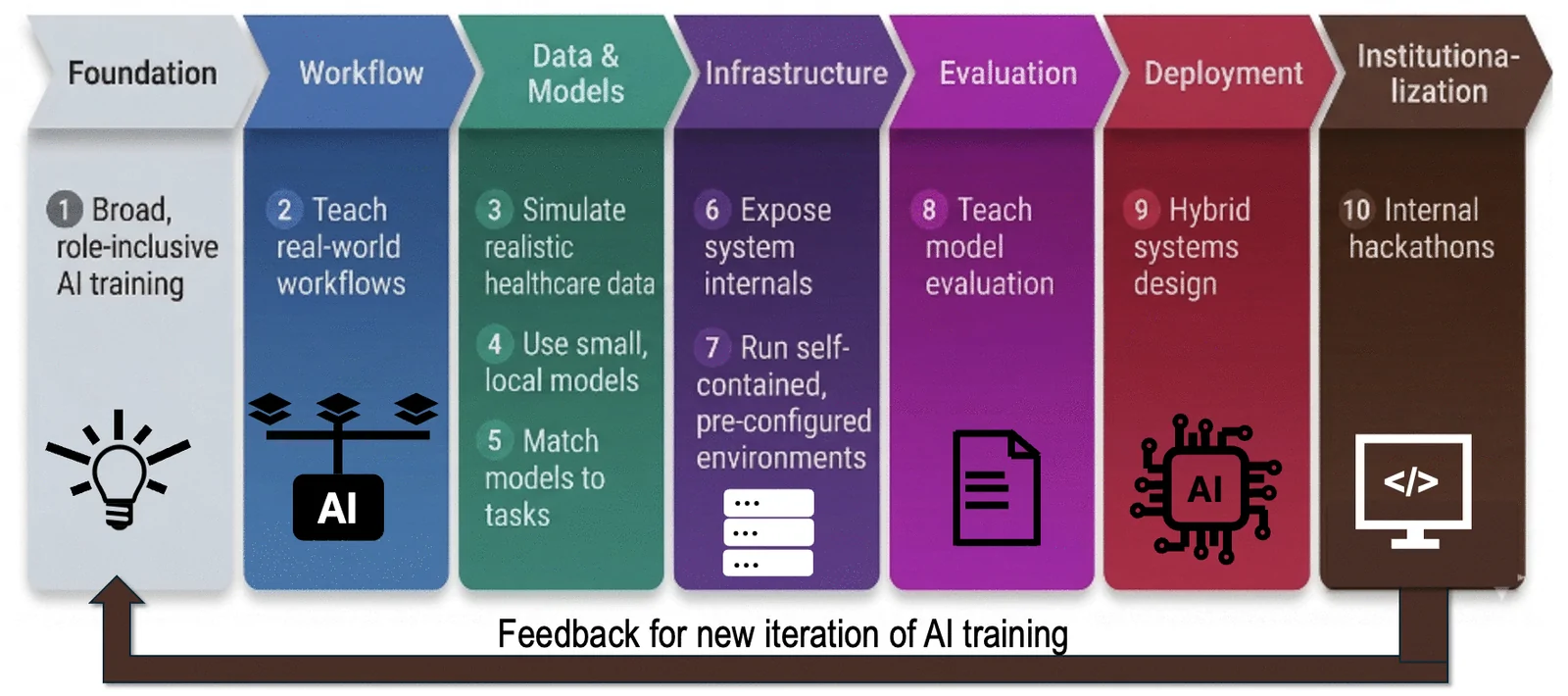
Ten simple rules for effective AI training for healthcare data team.

These rules are drawn from a two-day, hands-on course, Privacy-First AI Systems for Healthcare, delivered to more than sixty data engineers, analysts, data scientists, and managers at a comprehensive cancer center. The intended audience is the healthcare data, analytics, and application teams and the management that directs them. These teams are central to institutional AI adoption: they hold the infrastructure and the analytic context on which AI systems depend, and they are the group that must learn to supply that context in the patterns AI requires, which differ from those of traditional reporting and analytics. Training of this kind is therefore best understood as one substantial step toward organizational AI-readiness. The material is technical, but it is pitched at the level of systems rather than syntax: several participants had limited coding experience and still left able to reason about how the components of an AI system fit together.

Each learner worked in a pre-provisioned environment containing (AWS cloud) synthetic example data, teaching notebooks, and the basic services required for the exercises, and installed the AI models and runtimes themselves. The course was paired with a hackathon in which teams applied the material to problems of their own choosing, challenging them to adapt what they had learned into other relevant workflows. The training has since undergone several iterations and been delivered to further healthcare learners. The syllabus of this course is available in S1 Table.

All models used during instruction were instructor-curated, pre-trained models; the emphasis was on understanding model types and learning how to select and evaluate them rather than training models from scratch. During the subsequent hackathon, learners applied that framework to independently evaluate models that had not been introduced in the course.

The course was organized around a single clinical throughline: the interpretation of breast cancer pathology reports, and specifically the extraction and interpretation of biomarker status. Alongside the technical exercises, the course material set out the history of this problem in cancer care and why AI is unusually well suited to helping data teams bring this information into their data assets and build meaningful workflows around it. Each rule below was taught against that example, and it recurs through the rules that follow, so that the ideas are anchored in an applied problem rather than in an abstraction.

## Rule 1: Provide broad, role-inclusive AI training to avoid the creation of AI silos

Many organizations implicitly treat AI as the exclusive domain of data scientists, reinforcing functional silos in which data engineers are viewed as SQL specialists and analysts as users of statistical or business intelligence tools. This framing is increasingly misaligned with reality. Modern AI systems depend on contributions across the full data lifecycle, and limiting AI education to a single role constrains both organizational learning and system effectiveness.

AI training should therefore be intentionally broad and role-inclusive. Data engineers and analysts play a critical role in building the data pipelines, semantic layers, and governance structures that provide context for analytics and agentic AI systems, in which a model sequences the steps of a multi-step, variable process. Treating AI as a shared capability improves collaboration, reduces fragmentation, and ensures that AI initiatives are grounded in robust data practices, positioning teams to support both current analytical workflows and future AI-driven applications. This also scopes the audience. These rules address the data team: the engineers, analysts, developers, and data scientists who build and maintain data infrastructure. It also includes the managers who set priorities, and leadership who align data and AI strategy with institutional strategy.

## Rule 2: Teach real-world workflows

AI in practice is a system, not a component. Educational programs should resist organizing curricula around isolated abstractions such as LLMs, chat-based interfaces, or agentic frameworks. While these constructs are useful for communication, they mask the fact that real-world AI applications emerge from coordinated systems that integrate data engineering, retrieval, modeling, orchestration, and evaluation.

Instead, instruction should be structured around end-to-end workflows: ingestion of raw data, extraction of structured signals, retrieval of relevant context, ranking or classification of candidates, synthesis of outputs, and evaluation of results. Framing AI this way establishes a stable mental model that remains valid even as specific models and tools change. It also mirrors how healthcare data teams already think about pipelines, making AI feel like an extension of existing practice rather than a disruption to it.

## Rule 3: Simulate realistic healthcare data conditions

AI education should begin with first principles using realistic, synthetic data. Healthcare data is inherently heterogeneous, multimodal, and fragmented across sources, formats, and levels of structure. Synthetic data enables free experimentation and data sharing without introducing privacy or compliance risks, making it essential for instructional settings. To be effective, however, synthetic datasets must reflect real-world conditions, including incomplete records, inconsistent structure, and variability across sources, rather than presenting artificially clean or fully harmonized data.

In our course, learners worked with clinical guidelines, synthetic pathology reports, and a synthetic genomics database. Together, these sources introduced controlled but meaningful fragmentation, allowing participants to encounter common challenges such as schema mismatch, partial information, and cross-source reconciliation. This approach supported hands-on learning while preserving privacy and helped ground AI instruction in the operational realities of healthcare data work.

## Rule 4: Use small, local models for privacy-first experimentation, and teach their limitations explicitly

Learning must be safe before it is powerful. In healthcare settings, protected health information (PHI) is pervasive, and early experimentation with AI should not require transmitting sensitive data to external services. Small, locally deployed models provide a practical and effective solution. By running entirely within controlled environments, such as local workstations or institutionally managed servers, teams can experiment with realistic data without triggering privacy, compliance, or governance barriers. This approach lowers institutional resistance to experimentation and enables hands-on learning that may otherwise be infeasible. Privacy-first experimentation is therefore not merely a technical convenience, but a foundational requirement for responsible AI education in healthcare.

It is worth separating two ideas that the phrase “small and local” tends to merge. Small is a claim about cost and hardware: a model of a few billion parameters runs on commodity infrastructure. Local is a claim about data movement: no protected information leaves a boundary the institution controls. Local deployment does not by itself satisfy privacy or compliance obligations, but because data never crosses the institutional boundary it is a considerably safer way to experiment and to develop workflows. Keeping data inside that boundary is a central requirement of secure computing environments, and local AI is an important means to that end. Local also does not imply a laptop: our own learners worked on centrally provisioned instances that were remote but self-contained, and an institution can meet the same requirement through managed virtual desktops, an on-premises server, or a private cloud tenancy. Training should be designed against whichever boundary already exists.

At the same time, educational programs must explicitly address the limitations of small, local models. While these models are valuable for learning and prototyping, they are not universally interchangeable with larger or more capable systems. Instruction should demonstrate where small models perform well and where they fail, including constraints in reasoning depth, domain coverage, and robustness. Structured comparisons between small and larger models help learners develop intuition about trade-offs and appropriate escalation strategies. This balanced framing prevents both overconfidence and ideological bias, reinforcing a core engineering principle: AI models are tools with context-dependent strengths and limitations, not ends in themselves.

## Rule 5: Teach learners how to choose the right model for each task

One of the most valuable conceptual shifts for healthcare data teams is that different classes of model do different jobs. Some models are built to represent and retrieve text (encoders); some to rank or classify the candidates presented to them (cross-encoders); and some to generate prose or structured text (decoder, or generative, models). For example, in a cancer registry workflow, an encoder may retrieve relevant pathology reports, a cross-encoder may rank candidate matches, and a decoder model may generate a structured summary. Generative models are the most familiar because they power chat interfaces, and that familiarity often leads teams to reach for them indiscriminately, while retrieval and ranking models are new concepts for most learners. A concrete example makes the division of labor clear. In a cancer registry workflow, an encoder retrieves the guidelines relevant to a case, a cross-encoder ranks those guidelines by relevance, and a generative model produces a summary of the pathology report grounded in the most relevant guidelines.

Teaching these distinctions reframes AI as a toolbox of specialized components rather than a monolithic system. This perspective aligns naturally with healthcare workflows, where retrieval, classification, and reporting are distinct tasks with different requirements. It also enables more efficient and interpretable system design.

## Rule 6: Expose system internals before abstraction layers

Learners should see the wires before they are hidden. High-level frameworks and orchestration tools can accelerate development, but they also obscure important details. Educational programs should begin with transparent, minimalist implementations, often in notebooks or simple scripts, before introducing abstractions.

Our course adopted a “breadboard” approach. Breadboards are experimental circuits that can be quickly rewired and are used for prototyping electronics. Our training adopted a similar ethos in showing all the code while allowing students to experiment and change the code to see the different outputs. When abstractions are introduced later, they are understood as productivity aids rather than black boxes. Even students who adopt or evaluate third-party products for production will be equipped with a deeper understanding, allowing for better alignment between tools and needs. Given that the ecosystem of third-party tools and models is rapidly evolving, a foundational understanding is necessary to evaluate and select appropriate technologies for specific use cases.

## Rule 7: Use self-contained, pre-configured lab environments

Reproducible, pre-configured environments are essential for effective instruction. These environments should include all required dependencies and data, and should be isolated from external services by default. Our lab environments left room for students to install AI runtimes, models, and third-party modules, but students do not have to worry about configuring Jupyter Notebook or PostgreSQL, for example.

Learners who cannot diagnose a failing system are not independent, so the exercises deliberately surface failure: extractions that return nothing, retrieval that misses an obvious document, output that is confidently wrong. Learners are asked to determine whether the model, the prompt, or the surrounding code is at fault, and to adjust accordingly. The aim during instruction is to spend that diagnostic attention on the new concepts rather than on configuring sixty local environments. The lab environments were not used for the hackathon: there, teams translated their new skills into their own environments, which is where the boundary described in Rule 4 has to be established.

Self-contained labs support privacy, reproducibility, and comparability across learners [4]. They also mirror the controlled environments in which healthcare AI systems are often deployed, reinforcing institutional realism alongside technical skill [5].

## Rule 8: Teach evaluation before optimization

Evaluation is frequently an afterthought in AI education, yet it is central to trust and safety in healthcare. Learners should be taught to define ground truth, sample outputs systematically, conduct human review, and analyze failure modes.

By foregrounding evaluation, programs counter the prevailing hype culture and instill scientific rigor. Optimization becomes a meaningful activity only once correctness and reliability are understood. Students should learn how different models may perform wildly different on specific tasks and how to set up the evaluation to choose the best-performing model in both performance and accuracy.

Evaluation criteria should be derived from the decision the output supports, not from the model in isolation. In the biomarker workflow that runs through the course, we explain the consequences of the output: inappropriate treatment, excess financial burden, missed opportunities for life-saving clinical trials. The evaluation metrics include both accuracy versus an answer rubric and latency. Students are encouraged to balance the end use with requirements to contextualize the best approach. Teaching evaluation as a workflow rather than as a fixed metric is what makes it transferable: in our hackathon (Rule 10), teams applied the same framework to problems we had never presented and evaluated dozens of models that had not appeared in the course versus their own use cases.

## Rule 9: Teach hybrid systems, not end-to-end AI

Purely end-to-end AI solutions are often brittle and poorly suited to healthcare settings. In practice, robust systems combine traditional programming logic, database queries, varied retrieval mechanisms, and AI models within a single workflow. Deterministic components are typically responsible for data validation, filtering, aggregation, and comparison, while AI models are applied selectively to tasks involving interpretation or extraction from unstructured data. Teaching hybrid architectures reflects operational reality and enables more reliable, transparent system design.

For example, in our multimodal genomics workflow, AI models are used to extract biomarker status from unstructured pathology reports, while structured genomics data are analyzed using conventional SQL queries. The results are then compared using explicit business logic to generate a concordance assessment. This pattern illustrates a broader principle: trust in healthcare AI emerges not from model performance alone, but from how AI outputs are integrated with established data systems, rule-based logic, and human review to support accountability and auditability. These are governance properties, and hybrid design is what makes them achievable: deterministic components can be audited directly, model outputs can be monitored against a defined ground truth, and the boundary between the two is where oversight and risk management are applied [6].

## Rule 10: Formalize opportunities for applied experimentation after instruction

Instruction alone is insufficient to produce lasting organizational capability. AI training programs should be followed by structured opportunities for learners to apply what they have learned to real institutional problems. Mechanisms such as protected project time, learner-defined use cases, and internal hackathons [7] help translate individual skill acquisition into shared, operational capacity. Our team held a hackathon, scoped to the same audience as the training 6 weeks after the training concluded. This provided the opportunity to apply the principles from class to more bespoke use-cases after a period of experimentation and further research.

For example, trainees applied RAG concepts learned during the course to develop an AI-powered immune-related adverse events (irAE) explorer. The tool incorporates a Guideline-Grounded RAG module to answer questions about irAE clinical management using current national guidelines, along with several data-analytics modules [8].

These opportunities should be intentionally lightweight, prioritizing exploration and iteration over rigid deliverables. When organizations provide both time and institutional support for experimentation, learning extends beyond the classroom and becomes embedded in everyday practice, enabling sustained adoption and continuous improvement.

Finally, these 10 rules could be providing feedback and reiterated as new AI training topics to a new batch of trainees.

## Discussion

Together, these 10 rules address technical foundations, data realism, privacy, system design, infrastructure, scientific rigor, and institutional impact. None are focused on transient tools or trends. Instead, they articulate a pedagogy that treats AI as an evolving set of techniques embedded within stable healthcare workflows and constraints.

By adopting this approach, educators and leaders can help healthcare data teams move beyond performative AI adoption toward building systems that are trustworthy, maintainable, and aligned with patient-centered outcomes.

Two risks deserve explicit attention. The first is the pace of model turnover: the specific models taught in our first cohort were superseded within months, and any curriculum organized around particular tools inherits that expiry date. The second follows from it: tool-centric education produces learners who can operate a product but lack the framework to evaluate what replaces it. What has proven durable is systems thinking. Data are ingested and structured, relevant context is retrieved and ranked, a model interprets or generates, results are evaluated against a defined standard, and deterministic logic and human review surround the whole. Many components in our own course have since been replaced; the systems we teach have not changed. Nor is that generality confined to healthcare: subsequent cohorts have included leaders and engineers from financial services, a domain with comparable constraints on moving sensitive data beyond institutional boundaries, and the same rules and system patterns structure the training there.

Teaching around a specific use case also has limitations. Some learners find it difficult to carry the concepts beyond the examples used in class. Prompting during instruction helps: asking learners to map each pattern onto a problem they are familiar with. However, Rule 10 is where this is really addressed. In our hackathon, we were pleasantly surprised with the breadth of use cases addressed and the clever application of the systems learned in the training for novel use cases. Some hackathon projects have since progressed to production-level systems.

Governance has been implicit throughout. While our training does not directly prescribe a governance regime, it develops the shared vocabulary, systems understanding, and practical skills teams need to participate meaningfully in its design and implementation. Effective AI governance depends not only on policy, but on understanding how monitoring, auditability, evaluation, human oversight, and risk management operate within real systems. As AI evolves, organizations will need governance that can evolve with it. Teams and leaders with a common systems framework are better positioned to inform policy, translate it into effective controls, and adapt those controls as models, tools, and risks change.

## Conclusion

AI education in healthcare must do more than introduce new tools; it must cultivate judgment, systems thinking, and institutional readiness. The ten rules presented here offer a practical framework for achieving that goal. By teaching workflows over models, embracing data reality, prioritizing privacy, and formalizing applied experimentation, organizations can build AI capability that endures beyond the current hype cycle and contributes meaningfully to healthcare delivery and research.

## Supporting information

S1 Table Curriculum of the AI training for healthcare data team. (DOCX)
