# Supplementary material for: Ten simple rules for teaching practical AI to health data teams
Source: PLoS Comput Biol. 2026 Oct 30;22(10):e1014915. doi: 10.1371/journal.pcbi.1014915 (PMC13722196; doi:10.1371/journal.pcbi.1014915)
Supplement: Supplementary file 1 [file pcbi.1014915.s001.docx]

**S1 Table: Curriculum of the AI Training for Healthcare Data Team**

| **Module** | **STAGE & KEY TOPICS** | **WHAT YOU CAN DO AFTERWARDS** |
| --- | --- | --- |
| **0** | **Introduction to Local AI** | |
| 0.1 | **Downloading models to your environment** Ollama install · pulling local models: Granite, Phi, Gemma · the JupyterLab environment and terminal | Have local models downloaded and running in your own environment. |
| 0.2 | **Learn how AI works**  Attention · MLP · parameter count · quantization · runtime engines · CUDA and llama.cpp | Grasp the key concepts and components that power language models. |
| **1** | **Build Your First AI System — The Chatbot** | |
| 1.1 | **Introducing models & systems**  The Generative Model Card · token prediction · what a  Model Card and a System Card are for | Understand why the Generative Model is the foundation of text generation. |
| 1.2 | **Access models in Python from an API**  The Ollama Python API · prompts and responses · timing  metadata · why generate() has no memory | Call local models programmatically and get reliable, structured responses. |
| 1.3 | **Controlling model output**  System prompts first · temperature · top-k and top-p · max tokens · picking values per task | Control the style, creativity and length of outputs with confidence. |
| 1.4 | **Conversation history & the chatbot**  Why models are stateless · the messages list · system/user/assistant roles · trimming and windowing · streaming | Build a functional chatbot with context and streaming replies. |
| **2** | **Document Processing** | |
| 2.1 | **What a guideline is, and what it looks like** Why clinical guidelines exist · authoritative sources ·  reviewing a real guideline · this course’s synthetic set · the shapes a PDF arrives in | Recognize guidelines, their purpose and appropriate sources. |
| 2.2 | **Reading the page as an image**  The Vision-Language Model Card: image + prompt in, text out · rasterizing a page · scoring extraction against an answerkey | Extract guidance from a scanned page and measure how faithful the result is. |
| 2.3 | **Docling for document processing**  Parse text-based PDFs · text, headings, tables and structure · routing documents to the right processor · batch extraction | Turn a mixed folder of PDFs into structured, machine- readable records. |
| 2.4 | **Into the database**  A PostgreSQL schema for guidelines · loading extracted records · what to store beside the text | Stand up a guidelines database the rest of the course queries. |
| **3** | **Retrieval-Augmented Generation (RAG)** | |
| 3.1 | **Sparse retrieval**  BM25 and keyword indexing with Whoosh · why a search for a gene symbol returns nothing when every guideline uses the clinical name | Build a keyword index and see exactly where it fails. |
| 3.2 | **Vectors, and a database that holds them** SapBERT embeddings · normalized vectors and the dot  product · pgvector in PostgreSQL | Embed clinical text and store it for similarity search. |
| 3.3 | **Dense retrieval**  Semantic search over the guideline set · where meaning  beats spelling | Retrieve by meaning rather than by exact term. |
| 3.4 | **Hybrid search with reranking**  Combining sparse and dense results · a cross-encoder  reranker · scoring and cutoffs | Rank candidates so the best passage is the one the model sees. |
| 3.5 | **A retrieval library**  Packaging the three strategies behind one interface | Reuse retrieval as a component instead of rewriting it. |
| 3.6 | **The RAG chatbot**  Day 1’s chatbot, given retrieval · grounding answers · citing the guideline behind each claim | Answer pathology questions from retrieved guidelines, with sources. |
| **4** | **Multimodal Data Analysis & Benchmarking** | |
| 4.1 | **Pathology data flow**  Extract IDs and report sections · a biomarker prompt and a local model call | Extract structured data from a report and assess biomarker status locally. |
| 4.2 | **Benchmarking small language models**  25 reports across the four in-class models · one prompt, one rubric, repeatable runs | Run a repeatable pipeline that tests several local models on one task. |
| 4.3 | **Scoring & leaderboards**  A 0–4 scoring rubric · leaderboard and speed plots · reading a heatmap of failures | Score outputs objectively and choose models on accuracy and speed. |
| 4.4 | **The genomics database**  Copy number variation · what gene amplification means · SQL against a populated database | Query genomic findings for the same patients the reports describe. |
| 4.5 | **Concordance reporting**  The narrative result versus the structured one · the amplification check · flagging disagreement | Produce a concordance report reconciling two independent sources. |
| 4.6 | **Named entity recognition**  The NER Model Card · schema-driven extraction with GLiNER2 · batch pipeline and timing | Extract biomarkers at scale with an NER model instead of a generative one. |
| **5** | **De-Identification & Safety** | |
| 5.1 | **PHI detection with a local NER model**  GLiNER2-Guardrails-PII-Multi · nineteen entity types: fourteen the model was trained on, five defined for this chart · section-by-section detection | Find names, dates, contacts and identifiers in a synthetic clinical note. |
| 5.2 | **Redaction & verification**  Reverse-order tag replacement · resolving overlapping spans · confirming every detected identifier was replaced | Redact detected identifiers consistently and verify the result. |
| 5.3 | **Screening with a guard model**  A small local guard model · flagging jailbreaks and toxic content in one pass | Screen prompts before they reach the model. |
| **6** | **Agents and the Model Context Protocol (MCP)** | |
| 6.1 | **The agentic loop**  Observe, decide, act, repeat · the loop written by hand in about fifteen lines · where the model’s judgement ends and your code begins | Write an agent loop from scratch and name every part of it. |
| 6.2 | **Tools over MCP**  The Model Context Protocol · stdio transport · a self- describing tool server · tool discovery at runtime · what MCP does not solve | Connect an agent to an MCP server and discover its available tools at runtime. |
| 6.3 | **Appendix: the async client**  What the synchronous toolbox hides · event loops · the MCP client written out in full | Read and modify the asynchronous client underneath the convenience wrapper. |
